# Facilitatory and inhibitory pain mechanisms are altered in patients with carpal tunnel syndrome

**DOI:** 10.1371/journal.pone.0183252

**Published:** 2017-08-30

**Authors:** Benjamin Soon, Bill Vicenzino, Annina B. Schmid, Michel W. Coppieters

**Affiliations:** 1 Centre of Clinical Research Excellence in Spinal Pain, Injury and Health, School of Health and Rehabilitation Sciences, The University of Queensland, Brisbane, Australia; 2 Singapore Institute of Technology, Singapore, Singapore; 3 Physiotherapy, School of Health and Rehabilitation Sciences, The University of Queensland, Brisbane, Australia; 4 Nuffield Department of Clinical Neurosciences, University of Oxford, Oxford, United Kingdom; 5 Amsterdam Movement Sciences, Department of Human Movement Sciences, Faculty of Behavioural and Movement Sciences, Vrije Universiteit Amsterdam, Amsterdam, The Netherlands; 6 Menzies Health Institute Queensland and School of Allied Health Sciences, Faculty of Health, Griffith University, Gold Coast Campus, Queensland, Australia; University of Ottawa, CANADA

## Abstract

Preliminary evidence from studies using quantitative sensory testing suggests the presence of central mechanisms in patients with carpal tunnel syndrome (CTS) as apparent by widespread hyperalgesia. Hallmarks of central mechanisms after nerve injuries include nociceptive facilitation and reduced endogenous pain inhibition. Methods to study nociceptive facilitation in CTS so far have been limited to quantitative sensory testing and the integrity of endogenous inhibition remains unexamined. The aim of this study was therefore to investigate changes in facilitatory and inhibitory processing in patients with CTS by studying hypersensitivity following experimentally induced pain (facilitatory mechanisms) and the efficacy of conditioned pain modulation (CPM, inhibitory mechanisms). Twenty-five patients with mild to moderate CTS and 25 age and sex matched control participants without CTS were recruited. Increased pain facilitation was evaluated via injection of hypertonic saline into the upper trapezius. Altered pain inhibition through CPM was investigated through cold water immersion of the foot as the conditioning stimulus and pressure pain threshold over the thenar and hypothenar eminence bilaterally as the test stimulus. The results demonstrated that patients with CTS showed a greater duration (p = 0.047), intensity (p = 0.044) and area (p = 0.012) of pain in response to experimentally induced pain in the upper trapezius and impaired CPM compared to the control participants (p = 0.006). Although typically considered to be driven by peripheral mechanisms, these findings indicate that CTS demonstrates characteristics of altered central processing with increased pain facilitation and reduced endogenous pain inhibition.

## Introduction

Carpal tunnel syndrome (CTS) is the most frequent peripheral entrapment neuropathy leading to numbness, paraesthesia, pain and eventually motor deficits [1]. The traditional view is that median nerve compression at the wrist will produce signs and symptoms in accordance with the classical median nerve distribution. This is not always the case as CTS is often associated with symptoms as well as evoked hypersensitivity outside the median nerve innervation territory [2–4]. Such spread of symptoms has been attributed to central sensitisation, which can include both altered facilitatory and inhibitory mechanisms [5].

Studies evaluating the presence of central facilitatory mechanisms (e.g. central sensitisation) in CTS have so far been limited to quantitative sensory testing (QST). Several studies have identified widespread mechanical and thermal hyperalgesia in patients with CTS [2, 3, 6]. Specifically, central mechanisms seem to be predominantly present in a subgroup of patients with extramedian spread of symptoms [7, 8]. Recent work however which strictly excluded patients with comorbidities such as neck and arm pain could not identify widespread altered thermal and mechanical pain thresholds upon QST [9] and only found widespread elevated pain ratings during thermal pain testing [10]. As such, the evidence for central facilitatory mechanisms in patients with CTS as measured with QST is conflicting. To examine facilitatory central pain mechanisms, evaluation of pain hypersensitivity following an injection with hypertonic saline has been used in other patient populations with widespread pain [11, 12]. These studies demonstrated an increased pain response following hypertonic saline injection. As the injection sites were away from the affected area, the augmented pain responses in patients with long standing musculoskeletal pain was suggested to be related to a central pain mechanism such as central sensitisation [5, 13]. Given the inconsistent results of QST in patients with CTS [3, 6, 10], an alternative approach of evaluating pain sensitivity using hypertonic saline injection may provide clearer evidence of possible central facilitatory pain mechanisms in these patients.

Widespread symptoms and signs could also be caused by altered inhibitory mechanisms. Endogenous inhibitory mechanisms can be evaluated by examining if a test pain stimulus can be modulated by a noxious conditioning stimulus applied at some remote body site. This is known as conditioned pain modulation (CPM) or “pain inhibits pain” [14].The efficacy of CPM is reduced in patients with chronic musculoskeletal pain [15, 16], as well as in patients with neuropathic pain such as painful diabetic neuropathy [17], chemotherapy-induced polyneuropathy [18] and postherpetic neuralgia [19]. However, the findings in systemic neuropathies might not translate to localised mild entrapment neuropathies, such as mild to moderate CTS. Two studies investigated CPM in patients with a focal entrapment neuropathy [20, 21]. One study found that patients with painful peripheral neuropathy resulting from trauma or surgery had a reduced pain induced inhibition of painful heat stimuli, but not painful mechanical stimuli [20]. The other study revealed that the efficacy of CPM was reduced in atypical, but not in classical trigeminal neuralgia [21]. A potential role of altered inhibitory central mechanisms as tested by CPM has not been examined before in patients with CTS.

In order to better understand the facilitatory and inhibitory mechanisms at play in patients with CTS, a more comprehensive study is required. In this study, pain facilitation was examined in patients with CTS by evaluating pain hypersensitivity following an injection of hypertonic saline [22], which will complement findings from previous studies using QST [3, 23]. In addition, a CPM paradigm using the cold pressor test [24] was employed to investigate the efficacy of inhibitory mechanisms in these patients. These experimental findings will also be correlated to clinical presentation of pain and symptoms severity of patients with CTS in this study.

Clinically, a better understanding of the potential contribution of central pain mechanisms in patients with CTS would facilitate the interpretation of diagnostic tests and may have implications for management. With the current evidence available from QST suggesting the presence of central mechanism [3, 7, 8, 10, 25], it was hypothesized that patients with CTS will exhibit increased pain responses to hypertonic saline injection and a depressed CPM compared to healthy controls.

## Methods

### Participants

Twenty-five patients with CTS were recruited from a pool of 124 patients that had responded to an advertisement in the local printed media. These 124 patients had clinical signs and symptoms consistent with CTS and were all electrodiagnostically tested to have CTS. Based on the order of recruitment, the first 25 volunteers that agreed to participate in this study were recruited. Another 25 healthy participants without CTS volunteered to participate in this study. Patients with CTS had to meet the clinical [26] and electrodiagnostic [27] criteria for mild (n = 5) or moderate (n = 20) CTS. Furthermore, CTS symptoms had to be present for at least two months. Participants were ineligible to participate if they had any other current upper limb or neck disorder, for which treatment was sought in the preceding two years, systematic diseases, pregnancy or trauma related CTS. The healthy control participants were age and sex matched to the patients with CTS. The Boston carpal tunnel syndrome questionnaire scores [28] were also collected for patients with CTS (Table 1). All participants in the study were recruited from the same local community.

**Table 1:** Characteristics of the participants.

	CTS	Control
	(n = 25)	(n = 25)
Age	56.1 (9.1)	53.5 (9.5)
Gender (female / male)	15/10	15/10
Duration of symptoms	52.6 (62.3)	N/A
BCTSQ-Symptoms	2.2 (0.5)	N/A
BCTSQ-Function	1.6 (0.6)	N/A

All data are reported as mean (SD), except for gender. Age in years; Duration of symptoms in month; BCTSQ: Boston carpal tunnel syndrome questionnaire; CTS: carpal tunnel syndrome; N/A: not applicable.

All participants received the pain facilitation protocol first followed by a 10 minutes rest interval before starting the pain inhibition protocol. This study was approved by the University of Queensland, medical research ethics committee and all participants provided informed written consent prior to participating.

### Pain facilitation

Augmented pain facilitation was studied by investigating the response to experimentally induced pain [29]. A single bolus of 1.0 ml sterile hypertonic saline (5%NaCl) was injected into the upper trapezius muscle at a point 15 mm above the midpoint of a line between the C7 spinous process and the acromion [30]. An ultrasound system (LOGIQ-i, GE Medical Systems; Little Chalfont, Bucks, UK) was used to provide visual feedback to verify that the tip of the needle was positioned within the middle portion of the muscle. Experimental pain was induced on the affected side (or most affected side in case of bilateral CTS (n = 22)) for patients with CTS. The upper trapezius muscle location was chosen as it is outside the affected median nerve territory and potential changes would thus reflect central rather than local mechanisms. For the controls, the experimental pain was induced in the upper trapezius muscle on the dominant side.

Following the injection, participants rated the intensity of the induced muscular pain on a numeric pain rating scale (NPRS), ranging from 0 (no pain) to 10 (worse pain imaginable). In addition, participants rated the size of the perceived area of pain by referring to a chart with 10 circles with diameters of 1 to 10 cm [31]. Both ratings were repeated at 1-minute intervals until the NPRS was rated as zero.

The McGill pain questionnaire [32] was administered at the end of the pain facilitation protocol to describe the quality of the pain experienced following the hypertonic saline injection. The pain rating index from the McGill pain questionnaire, which is the sum of the numerical values given to each pain descriptor, was used in the analysis [33].

### Conditioned pain modulation (CPM)

A CPM paradigm with the cold pressor test [34] was used to evaluate the efficacy of the pain inhibitory system. Participants submerged one foot into an insulated container filled with cold water with the medial malleolus ~3 cm below the water line. The foot contralateral to the affected hand (or most affected hand in case of bilateral CTS) was submerged. Participants rated the cold-induced foot pain on a NPRS, ranging from 0 (no pain) to 10 (worst pain imaginable) [33]. The water temperature at the start of the experiment was set at ~10 degrees Celsius but was modified if needed to ensure that a predetermined target pain intensity (i.e., a NPRS score between 4 and 7) was reached. A digital thermometer was placed in the insulated container to measure the water temperature. Upon submerging the foot, the participants were asked to rate the NPRS score at intervals of 10 seconds. More ice or water was added into the container to adjust the water temperature and achieve the target NPRS score between 4 to 7. Immediately after maintaining the NPRS within the target score for 30 seconds, the test stimulus was given. This was done to ensure that participants could tolerate the conditioning stimulus and complete the experiment.

Pressure pain threshold (PPT) testing was applied as a test stimulus to determine potential CPM effects during the cold water immersion. PPTs were measured with a digital algometer (Somedic AB, Farsta, Sweden) by applying pressure through a 1-cm^2^ rubber plate at a constant rate of 40kPa per second on the thenar and hypothenar eminence of both hands. The participants pressed a button as soon as the sensation of pressure changed to pain. PPT measurements were taken on the thenar and hypothenar eminence of both hands, before cold water immersion and during cold water immersion. The less severe or non-dominant hand was measured first and all PPT measurements were repeated three times for each location. The mean values of the three repetitions were used for analysis. Participants were asked to rate the NPRS for the immersed foot after the PPT measurements at each location. If the rating fell out of the targeted range (NPRS 4–7), the water temperature was adjusted accordingly before commencing PPT measurement at the next location. NPRS was used as a control to standardise the cold pressor test instead of submersion time because it provided the better estimate of the pain stimulus.

The healthy control participants underwent the same CPM protocol with the foot contralateral to their dominant hand submerged in the cold water bath during the experiment.

### Statistical analysis

For the pain facilitation protocol, the NPRS, the size of the perceived area of pain, the duration of pain, and McGill questionnaire pain scores were compared between groups using Student’s t-tests. Results of NPRS and size of perceived area of pain were plotted against time and the area under the curve of the plotted graphs was compared using Student’s t-tests.

For CPM, the effect of pain modulation was expressed as the change in PPT values from before cold water immersion to during cold water immersion for each location. In addition, relative differences in PPT were calculated as a percentage of change to the baseline PPT measured at each location. A two-way ANOVA was applied to both measurement of PPT changes to determine the difference in the effect of CPM between GROUPS (CTS vs control) and LOCATIONS (thenar, hypothenar on left and right hand).

Pearson’s correlation analysis (2-tailed) was done to examine the relationship between the experimental data from pain facilitation and inhibition protocol to clinical presentation of pain and symptoms severity in patients with CTS. Shapiro-Wilk test was used to determine normality of the data. All analyses were performed in SPSS, version 23 (SPSS Inc, Chicago, IL, USA) with the level of significance set at p<0.05.

## Results

### Pain facilitation

The pain ratings following the hypertonic saline injection for patients with CTS and the control group are summarised in Table 2. In response to experimentally induced muscle pain, patients with CTS reported a higher pain intensity (mean = 49.1, SD = 20.0) than the control group (mean = 38.6, SD = 15.5); t(48) = 2.06, (p = 0.044). A larger perceived area of pain was reported by patients with CTS (mean = 54.5, SD = 16.4) compared to the control group (mean = 40.3, SD = 21.5); t(48) = 2.62, (p = 0.012). In addition, the pain lasted longer in patients with CTS (mean = 11.4, SD = 2.9) than the control group (mean = 9.7, SD = 3.17); t(48) = 2.03 (p = 0.047). There was no difference between patients with CTS (mean = 17.8, SD = 9.77) and controls (mean = 17.8, SD = 8.62); t(48) = -0.03, (p = 0.976) for the pain rating index of the McGill pain questionnaire. All data were normally distributed.

**Table 2:** Pain ratings following hypertonic saline injection.

Main group analysis: CTS versus control	CTS (n = 25)	Control (n = 25)	Mean difference between groups (95% CI)	p-value
Pain intensity (NPRS)	49.1 (20.0)	38.6 (15.5)	10.4 (0.3 to 20.6)	0.044
Size of perceived area of pain	54.5 (16.4)	40.3 (21.5)	14.2 (3.3 to 25.0)	0.012
Duration of pain (in mins)	11.4 (2.9)	9.6 (3.2)	1.8 (0.02 to 3.5)	0.047
McGill pain questionnaire (PRI)	17.8 (9.7)	17.8 (8.6)	1.2 (-0.5 to 7.4)	0.976

All data reported as mean (SD), unless otherwise indicated. NPRS: numeric pain rating scale (area under the curve); Size of perceived area of pain: circle rating scale (area under curve); PRI: pain rating index

### Conditioned pain modulation

**Fig 1:**
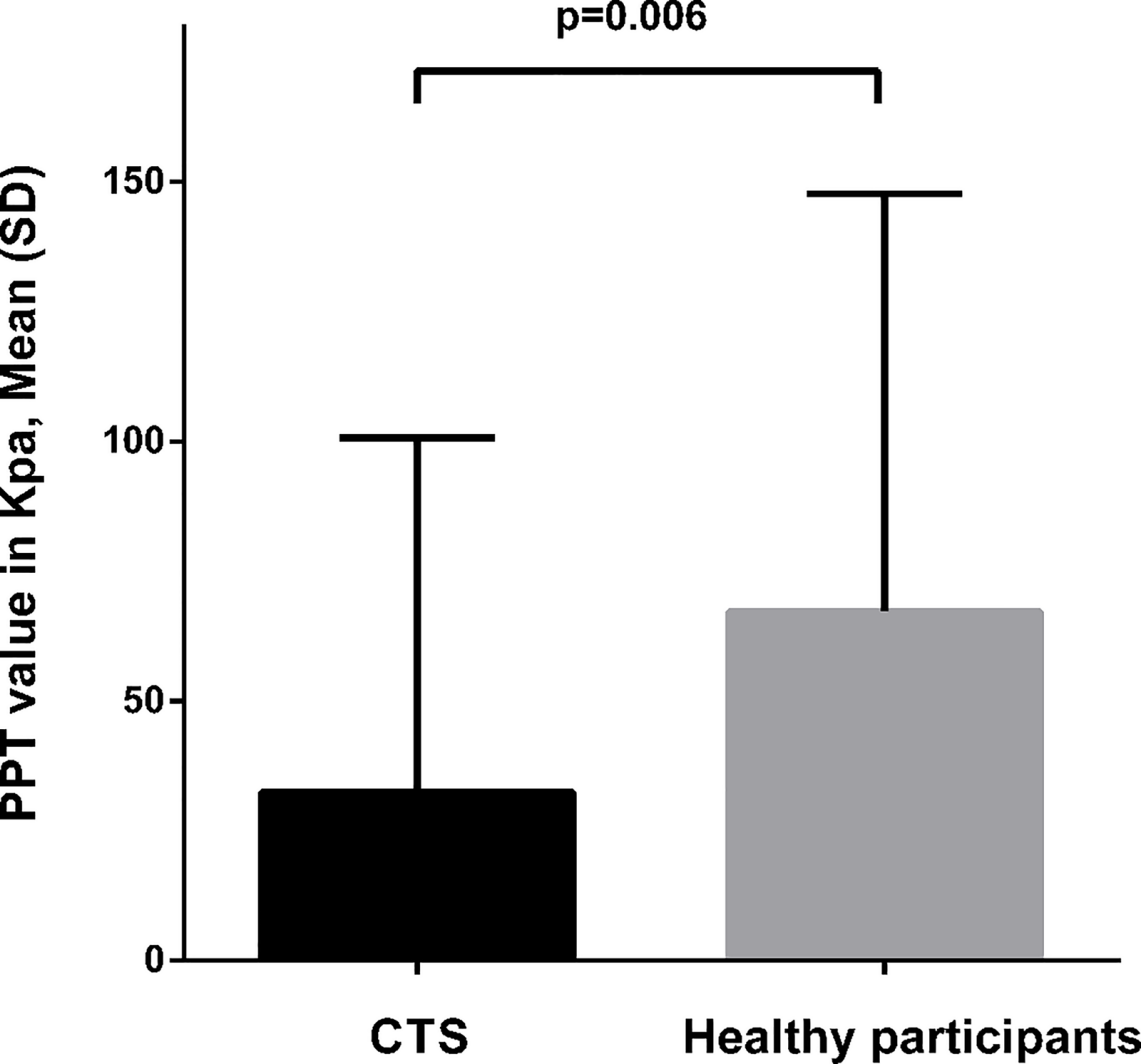
Efficacy of CPM. Results of univariate analysis for difference in pressure pain thresholds (PPT) pre and during cold water immersion.

Table 3 summarises the PPT values for the CPM paradigm for the patients with CTS and the control group. Analysis using difference in PPT values revealed a main GROUP effect (p = 0.006; CTS mean = 32.4 kPa, SD = 77.1, Control mean = 67.2 kPa, SD = 97.6, Fig 1). No main effect for LOCATIONS (p = 0.985) or GROUP X LOCATIONS interaction effects (p = 0.722) were found. Analysis using percentage changes to PPT values showed no main GROUP effect (p = 0.096), LOCATIONS (p = 0.88) or GROUP x LOCATIONS interaction effect (p = 0.51). This difference in the results of the two PPT findings may indicate a potential difference in baseline PPT between groups. Additional analysis was conducted to compare PPT measurements at baseline between groups. However, the results of PPT comparison at baseline showed no significant difference between patients with CTS (mean = 487.7, SD = 206.9) and controls (mean = 478.4, SD = 137.4); t(198) = 0.372 (p = 0.71). All data were normally distributed.

**Table 3:** Pressure pain thresholds before and during the cold pressor test.

Main group analysis:		CTS		Control		CTS	Control
CTS vs control							
		Before	During	Before	During	CPM efficacy	CPM efficacy
						*Percentage difference*	*Percentage difference*
1	Thenar	475.2 (204.9)	506.5 (192.2)	459.5 (129.4)	533.9 (162.2)	31.2 (63.1)	74.3 (91.8)
						*0*.*095 (0*.*15****)***	*0*.*172 (0*.*20)*
	Hypothenar	501.9 (213.3)	543.9 (199.9)	520.2 (119.4)	579.2 (211.9)	41.9 (85.9)	59.1 (115.4)
						*0*.*127 (0*.*20)*	*0*.*107 (0*.*18)*
2	Thenar	451.4 (166.0)	488.8 (144.4)	450.5 (122.4)	513.1 (129.7)	37.4 (69.0)	62.5 (85.5)
						*0*.*117 (0*.*18)*	*0*.*158 (0*.*20)*
	Hypothenar	522.1 (241.9)	541.2 (227.4)	483.4 (143.9)	556.5 (189.5)	19.1 (89.9)	73.0 (100.2)
						*0*.*072 (0*.*19)*	*0*.*151 (0*.*20)*

Pressure pain thresholds in kPa; data reported as mean (SD). CTS: carpal tunnel syndrome; 1: affected (or most affected) hand for CTS; dominant side for control; 2: unaffected (or least affected) hand for CTS; non-dominant for control; CPM efficacy: difference between PPT measurement before and during cold pressor test. Italic values: relative changes in PPT

The results of the Pearson correlation analysis showed that none of the experimental data are correlated to the results of McGill pain questionnaire, BCTSQ or the duration of symptoms.

## Discussion

The study revealed that patients with mild to moderate CTS demonstrate altered facilitatory and inhibitory pain processing that are typically linked to central sensitisation [5]. Evidence of central pain mechanisms accounting for hypersensitivity was apparent in patients with CTS as the saline injection was administered at a site remote from the symptomatic area in the hand [1]. This remote increase in pain hypersensitivity in the upper shoulder region is therefore unlikely to be attributed to focal mechanisms in the carpal tunnel. This study also demonstrated a reduced efficacy of CPM in patients with CTS. Our findings add to the small, but growing body of evidence [3, 10, 35–38] that central pain mechanisms contribute to the clinical presentation of CTS and possibly play a role in its diagnosis and management.

CPM reflects the activity of the descending endogenous analgesic system [39] and has been used to investigate changes in central pain processing [40]. Our findings on CPM in patients with CTS suggest that these patients share similar changes in central pain processing with conditions for which widespread pain hypersensitivity and impaired CPM is well established, such as fibromyalgia [41], painful knee osteoarthritis [42, 43] and chronic tension-type headache [44]. It is interesting that changes in CPM occurred in patients with mild to moderate CTS in our study, because, unlike some conditions with persistent pain states, CTS is typically not characterised by high pain intensity levels [10]. It must be noted that although the difference in PPT results was significantly decrease in patients with CTS indicating a reduction in efficacy of CPM, the percentage changes in the PPT did not differ between groups. Since baseline PPT values were comparable, the data is still more likely to reflect a reduction in CPM efficacy in patients with CTS compared to the control.

Our patients had BCTSQ scores that were generally less severe than patients with CTS requiring surgical intervention [45] and we only included patients with mild to moderate changes upon electrodiagnostic testing. It would seem from our data that changes in endogenous pain modulatory mechanisms in peripheral neuropathy such as CTS are not entirely dependent on the extent of neurological damage or symptom severity. Since the efficacy of CPM varies even in healthy participants, it has been argued that a pre-existing impaired CPM capacity may be a risk factor to develop acute or chronic pain conditions rather than an effect of the injury or disease [46]. Future longitudinal studies are however warranted to evaluate this hypothesis.

The results from the hypertonic saline injection paradigm revealed that the induced pain in patients with CTS was more intense, lasted longer and was felt in a larger area compared to matched control participants. Our findings are in line with other studies using hypertonic saline injection in different patient populations (e.g., patients with whiplash or non-traumatic neck and shoulder pain), which have also shown a larger area [13, 47] and intensity of pain [47] at an injected site distant to their symptoms. This extensive spread of pain and higher pain intensity shown by patients with CTS following the hypertonic saline injection are similar to other musculoskeletal conditions in which evidence of pain hypersensitivity are well established [13, 43, 48]. Since such remote pain enhancement in patients with CTS cannot solely be ascribed to peripheral mechanisms at the wrist, our results may reflect changes in sensory processing more proximal in the nociceptive pathway such as in the dorsal root ganglia, spinal cord or higher centres [5].

Comorbidities, such as neck pain and arm pain, are common in patients with CTS [4]. These conditions may interact in a complex way to modulate pain and hypersensitivity. It has previously been shown that the strict exclusion of co-morbidities such as neck or arm pain counters the assertion that patients with CTS exhibit a spread of hyperalgesia [9, 10]. In the current study, we only included patients with CTS symptoms limited to the hand in order to specifically evaluate central processing without confounding factors. Interestingly, the current data suggest that despite the absence of comorbidities, patients with CTS show signs of altered central processing as apparent by a more pronounced reaction to remote hypertonic saline injection and reduced CPM efficacy. These findings indicate that patients with CTS have changes in pain processing that are typically linked to central pain mechanisms. Whereas these changes may be intimately linked with continuous altered peripheral input from the compression site, it could clinically be warranted to consider pharmacological and therapeutic pain management strategies beyond the local compression site in the management of those patients with predominant central changes.

The results from Pearson’s correlation analysis revealed no significant correlation findings between clinical scores from McGill pain questionnaire, BCTSQ, duration of symptoms and all pain experimental data. We suggest that a study powered specifically for correlation analysis may be needed to evaluate this relationship further.

The cross sectional nature of this study limits the ability to determine CTS as the cause or result of altered central processing. Other factors not within the scope of this study (e.g., psychological or genetic factors) could influence the susceptibility of our participants to exhibit changes in central processing before the development of CTS. Designing a study to determine this causational effect will however be methodologically challenging.

## Conclusion

This study investigated pain facilitation and inhibition in patients with mild to moderate CTS. Remote hypertonic saline injection and CPM testing demonstrated characteristics of altered central pain processes in patients with CTS via increased pain facilitation and reduced endogenous pain inhibition.
